# The Impact of Family Care for the Elderly on Women’s Employment from the Perspective of Bargaining Power

**DOI:** 10.3390/ijerph18115905

**Published:** 2021-05-31

**Authors:** Yujuan Huang, Haoying Xu, Hengyu Liu, Wenguang Yu, Xinliang Yu

**Affiliations:** 1 School of Science, Shandong Jiaotong University, Jinan 250357, China; 211018@sdjtu.edu.cn; 2 School of Insurance, Shandong University of Finance and Economics, Jinan 250014, China; xuhaoying1996@163.com (H.X.); 20163433@sdufe.edu.cn (X.Y.); 3 Thurgood Marshall College, University of San Diego, San Diego, CA 92092, USA; hel107@ucsd.edu

**Keywords:** family elderly care, women’s employment, bargaining power, heterogeneity, endogeneity

## Abstract

Due to the wishes of the elderly and the traditional family culture in China, family care is the main way of providing for the aged, and women’s care is the main way. This is not conducive to the protection of women’s employment rights and the realization of self-worth under the background of increasing women’s autonomy. Based on the latest data of the China Health and Nutrition Survey Database (CHNS), this paper uses ordinary least squares (OLS) and the instrumental variable method of control endogeneity to analyze the influence of family care activities on the labor participation rate of married women. The innovation of this paper is to introduce family bargaining power into this kind of model for the first time, and further analyze the heterogeneity from the perspective of bargaining power differences. The empirical results show that the family elderly care activities have an obstacle effect on married women’s participation in employment, and the family members with strong bargaining power will significantly hinder employment, so this paper puts forward policy recommendations in line with the actual situation, hoping to provide theoretical support for the improvement of the social security system for the elderly.

## 1. Introduction

China’s population aging problem has become more prominent, and people’s demand for elderly care services is also growing. According to statistics of the State Bureau of statistics, at the end of 2019, the population aged 0–15 years old was 24,977 million, accounting for 17.8% of the total population; the population aged 16–59 was 896.4 million, accounting for 64.0%; the population aged 60 and over was 25,388,000, accounting for 18.1%, of which, the population aged 65 and over was 17603 million, accounting for 12.6%, far exceeding 7% of the international population aging standard. At present, China’s aging is facing the following problems: the large base of the elderly population leads to the rapid growth of the aging population; the proportion of the elderly living alone and empty nesters increases, and the growth speed is accelerated; the aging of the population makes the problem of family care for the aged more and more serious.

With the increasing aging problem, the demand for nursing care of the elderly also increases. Although the function of family support is gradually declining, due to the influence of traditional Confucian culture, China advocates filial piety. Since ancient times, the tradition is to support the elderly by family children. Through the demand theory, we can realize the unique advantages of family care for the elderly to meet the spiritual and cultural needs of the elderly, and realize that family care for the elderly is an indispensable part of the society, so family care still plays a leading role in elderly care. Family care for the elderly refers to the free care provided by the family members of the elderly (spouse and children, etc.). According to the third follow-up survey of China’s elderly population, 84% of China’s elderly population rely on family care to meet the needs of daily life. The increasing demand for family care caused by the aging of population is mainly reflected in two aspects: first, the family planning policy implemented since 1982 has reduced the number of new family members, led to the gradual transformation of family structure to miniaturization, increased the pressure of family members to support the elderly, and increased the dependency ratio of the elderly; second, the improvement of medical and living conditions has made the elderly population older, and the demand for family care has further increased. At present, all over the country, initiatives are carrying out pilot projects on family pension beds, developing social pension services. At the key node of pension from family to society, the research on the impact of family elderly care on women’s employment is particularly important, which can provide reasonable suggestions for the current pension environment which is lacking spiritual and cultural services and uneven quality of attendants.

In most traditional families, if considered from the perspective of family economics, the labor distribution among family members is mainly as follows: men are mainly responsible for hunting, farming and other strong physical labor, while women are responsible for giving birth to children and handling routine household chores. This division of labor standard is mainly based on the gender, physiology, good at the project, which can effectively increase the satisfaction of family members. Since ancient times, there has been a traditional idea that “men dominate the outside and women dominate the inside”. Under the influence of feudal ideology, women are often responsible for family work. However, due to the continuous development of social economy, more and more highly educated women appear in the workplace, and the gender differences between men and women are further weakened. Compared with male employees, women also have their own value and competitiveness in the labor market. Especially in family life, the changes caused by gender differences have been changed. However, in real life, most women are still the daily life managers of family members, and family care still occupies most of women’s energy and time. Therefore, the purpose of this paper is to set women as the main undertakers of family care for the elderly by combing the theory of family economics and introducing the national conditions. This is not only conducive to the accurate evaluation of married women’s contribution and contribution in family elderly care activities, but also conducive to the in-depth discussion of the relationship between the current population aging situation and women’s social status, so as to formulate positive and effective policies under the background of population aging and the policy of “opening two children in an all-round way”.

The trend of population aging intensifies the conflict between taking care of parents and employment in children’s time allocation, and further deepens the contradiction between taking care of children and employment. Due to the increasing opportunity cost of family care for the elderly, children are suffering from the expanding implicit “wage punishment” to care for the elderly. However, due to the different resource allocation preferences and maintenance responsibilities of husband and wife, they will make different decisions in the redistribution of family resources. Based on this, this paper analyzes the impact of family elderly care on married women’s employment under the premise of increasing time cost from the perspective of total sample and bargaining power difference. Based on the latest panel data of CHNS, this paper uses the instrumental variable method to provide reference measures for solving endogenous problems, to investigate the hindering effect of family elderly care on married women’s labor participation rate, and to further explore the difference of this effect in bargaining power.

Under the background of aging population and miniaturization of family structure, the relationship between family care and labor participation rate has become an important research content of many scholars at home and abroad in recent years. The research on this aspect started from Soldo and Myllyluoma (1983), and most of the previous studies were small-scale sample analyses [1]. This paper is the first one to use the national representative database to study the relationship between family elderly care and caregiver employment. Stone and Short (1990), Carmichael and Charles (1998), Lilly et al. (2010) all studied the relationship between care activities and labor participation rate, and gave some ideas and opinions [2,3,4]. Although previous studies assumed that family care for the elderly is exogenous relative to employment decision-making, resulting in the lack of control to deal with endogenous problems, resulting in biased and inconsistent estimation results, these research results have a profound impact on the understanding of the relationship between family care for the elderly and employment rate. In recent years, under the background of the rapid promotion of simultaneous equations, instrumental variables and panel data, in order to find the real impact of family elderly care on children’s labor participation, using rigorous measurement methods to control the endogeneity of the two has become the focus and difficulty of research. Casado-Marín et al. (2011), based on the data of the European community household survey, used the method of dynamic panel fixed effect analysis to explore the influence of different types of family care and care hours on labor time, and used the dynamic ordered profit model to control the endogenous problems caused by the characteristic differences of unobservable individuals. The empirical results show that women with a high intensity of care, living with their parents and providing family care for the elderly for a long time, have a significant negative impact on employment [5].

Due to the profound influence of the Confucian theory based on filial piety and the traditional idea of patriarchy, family care plays an important role in the elderly care. The empirical analysis results of Su et al. (2015) show that more than 90% of the disabled elderly mainly rely on family care, with less forms of social care [6]. Ma (2013) pointed out that female family members play a leading role in the care of the elderly, and the state constantly emphasizes the importance of family support from the legal and policy level. At the same time, with the development of the times, the voice of advocating gender equality becomes increasingly loud [7]. Therefore, the discussion on the relationship between family care and women’s labor participation in China has become increasingly in-depth in recent years. Liu et al. (2010) used the two-stage predictive substitution method to solve the endogenous problem of the model, and analyzed the influence of taking care of parents on the employment of urban married women [8]. Due to the advantages of the instrumental variable method in controlling endogeneity, most papers adopt this method, such as Liu (2014), Fan and Chen (2015), Chen et al. (2016), Wu et al. (2017), Guo and Jiang (2019), Fan and Xin (2019), Song (2019), Zhang and Xu (2020). Gu (2021) used the CLHLS database and its paired data, the CHNS database, and the CFPS database as samples to study the impact of family care on children’s labor participation and the impact of gender differences on this issue. When they dealt with the data, they all used the instrumental variable method to control endogenous problems [9,10,11,12,13,14,15,16,17]. Chen and Dong (2011) found that the elderly with higher socioeconomic status get more care time and cash help from their children [18]. Ning and Luo (2015) studied the influence of the difference of bargaining power between husband and wife on the elderly care behavior of rural families [19]. The conclusion of the above papers shows that the difference of bargaining power between husband and wife has an impact on care behavior. Qi (2005) used the Nash bargaining model to discuss the definition and measurement method of bargaining power, and found that the difference of bargaining power has a certain impact on family internal problems, so bargaining power has a certain role in family elderly care activities as an important family problem [20].

To sum up, although the domestic research on the relationship between caring for the elderly and employment has begun in recent years, most of the literature is limited to the gender and urban-rural differences of children, mainly on the basis of individuals, and there is no research on the impact of family care on children’s employment from the perspective of family bargaining power. The innovation of this paper is to introduce family bargaining power into this kind of model for the first time, and further analyze the heterogeneity from the perspective of bargaining power differences.

## 2. Model Construction, Data Source and Variable Description

### 2.1. The Construction of Model

As an important resource, time plays an important role in the allocation of family resources. Family members’ time can be divided into three parts: working time, family working time and leisure time. The individual allocates this scarce resource with the goal of maximizing the utility. Therefore, family elderly care, as an important family work, will inevitably hinder family members from going out to work. The individual allocates this scarce resource with the goal of maximizing utility, so family care for the elderly as an important family work will inevitably hinder family members from going out to work. Through the theory of family economics to understand the conflict between family elderly care and work, and connecting with the previous research results that family care for the elderly hinders children’s employment, this paper establishes the research hypothesis that family elderly care has a negative impact on women’s employment.

The purpose of family members’ behavior is to maximize the “Nash social welfare”. We use the Nash bargaining model that is often used to analyze the decision-making behavior of family members to briefly describe the role of bargaining power on family care. The utility function is used to measure the degree of personal satisfaction from consumption to reflect the impact of the consumption combination of work, family labor and leisure on personal satisfaction. In this paper, the effect of leisure on utility is not discussed, then the individual utility function is defined as

(1) $$U^{i}=U^{i}(C^{i},L^{m},L^{f},H^{m},H^{f},\beta^{i})$$

where, C represents consumption, L represents the vector of family public goods provided by husband and wife, including the care for parents’ health and welfare and the provision of other public goods. H is labor production input, β is preference of supporting parents, superscript m is male, superscript f is female, and superscript i is gender according to specific situation. Based on the above hypothesis, the maximum utility of an individual in the family can be expressed as

(2) $$\left\{ \begin{array}{l} \mathrm{Max}(U^{m}-V_{0}^{m})(U^{f}-V_{0}^{f})\\ s.t.\;\;\sum I^{i}+\sum p_{w}^{i}T^{i}=\sum Cp_{C}^{i}+\sum p_{w}^{i}(L^{i}+H^{i}) \end{array}\right.$$

where, $V_{0}$ represents the bottom line, U is the maximum utility in Equation (1), $p_{C}$ is the price vector of consumer goods, $p_{w}$ is the wage rate, I is the non-labor income, and T=L+H is the total investment. According to Equation (2), bargaining power is determined by the bottom line. In this model, the maximum utility that family members can obtain outside the family is the bottom line, that is, the bottom line is determined by consumption and labor input.

(3) $$\left\{ \begin{array}{l} \mathrm{Max}U^{i}(C^{i},L^{i})\\ s.t.\;\;I^{i}+p_{w}^{i}T=C^{i}p_{C}^{i}+L^{i}p_{w}^{i} \end{array}\right.$$

Equation (3) is the individual utility function under the given consumption combination of consumption and labor production input. It can be seen that consumption is determined by the non-labor income and the price vector of consumer goods, and the labor production input is expressed by the wage rate.

(4) $$V_{0}^{i}=V_{0}^{i}(I^{i},p_{w}^{i},p_{C}^{i})$$

Equation (4) is the influencing factor analysis of the bottom line, from which we can see that the bottom line is determined by the non-labor income, wage rate and the price vector of consumer goods. Therefore, it is reasonable and feasible to use the wage rate to express the bargaining power when other conditions are unknown or unchanged.

According to the Nash bargaining model, the bottom line and bargaining power are determined by the maximum utility of individuals outside the family, which have an impact on the allocation of resources within the family. Family members’ endowment support to parents may be related to the difference of bargaining power between family members and their spouses in the family. If their bargaining power is higher than that of their spouses, then children are more likely to have the right to decide the form and quantity of endowment support to their parents. As an important family resource allocation problem, the bargaining power plays a certain role in the impact of family care for the elderly on women’s employment.

Based on the gender perspective, this paper analyzes that the imbalance of time and energy distribution in the current society has an impact on the labor participation and family care of men and women, which may make it difficult for individuals to maintain the balance between work and family. This paper uses panel data to study the hindering effect of elderly care on married women’s employment, and its construction of model is as follows

(5) $$Y_{it}=\alpha_{0}+\alpha_{1}Care_{it}+\alpha_{2}X_{it}^{1}+\alpha_{3}X_{it}^{2}+\mu_{i}+year_{t}+\varepsilon_{it}$$

where, the dependent variable $Y_{it}$ indicates whether married women i take part in work at time t, i=1 indicates taking part in work, and i=0 indicates not taking part in work. The important explanatory variable $Care_{it}$ indicates whether married women take care of their parents (in laws), i=1 indicates that they take care of their parents (in-laws), and i=0 indicates that they do not take care of their parents (in-laws). $X_{it}^{1}$ indicates the personal characteristics of married women i at time t, including age, urban-rural household registration, and their own health. $X_{it}^{2}$ refers to the family characteristics of married woman i at time t, including bargaining power, whether to care for children under 6 years old, number of family members, and whether to live with parents or in-laws. $\mu_{i}$ is the individual fixed effect; $year_{t}$ is the time fixed effect; $\varepsilon_{it}$ is a random error term. A detailed explanation will be given in the model selection section below.

Due to the endogeneity caused by the two-way causal relationship between participation in work and care for the elderly, it is impossible to judge whether it is because of caring for the elderly and unable to participate in work or because of insufficient ability and unable to enter the labor market. Therefore, we solve the endogeneity problem through the instrumental variable method. This paper takes the number of brothers and sisters and whether parents (in-laws) need care as instrumental variables. These two variables have no impact on the employment participation rate, but have an impact on whether to care for the elderly, meeting the requirements of instrumental variables. Therefore, the idea of this paper is to first use the ordinary least squares (OLS) to analyze the impact of family care activities on the employment rate of married women, and then, further use the instrumental variable method to control endogeneity for better estimation, and then, carry out a differential analysis from the bargaining power level to identify the role of this variable.

### 2.2. Data Source and Variable Description

The data in this paper come from the China Health and Nutrition Survey (CHNS). The database covers the survey data of Beijing, Shanghai and Chongqing, Guangxi Zhuang Autonomous Region, and 11 provincial administrative regions of Shandong, Liaoning, Heilongjiang, Jiangsu, Zhejiang, Henan, Hubei, Hunan, Shaanxi, Yunnan and Guizhou. The sample time span of this paper is 1991–2015, involving women under 52 years old. The variables related to family elderly care in this study were all from the “married women” questionnaire, and the basic information was from the “demographic” and “work and income” questionnaires. After removing key missing variables, the final sample information included 1728 married women.

The dependent variable “employment situation” in this paper comes from “do you have a job now?” in the “work and income situation” questionnaire of the CHNS database, where, 0 means no work, 1 means to participate in labor. It can be seen from Table 1 that among the 1728 respondents, there are 1406 married women who are working and 297 who are not. The average employment participation rate of women is 82.6%. The average employment rate of married women who are engaged in family care for the elderly is 78%, which is lower than that of women who are not engaged in family care for the elderly by 3.9%. The core explanatory variable “whether to take care of parents” comes from the “relationship with parents” questionnaire. Herein, 1 means that they took care of his/her daily life or accompanied him/her to go shopping last week, 0 means that they did not. A total of 778 people bear the responsibility of family elderly care, 925 people do not bear the responsibility of care. In the total sample, married women engaged in family elderly care accounted for 46.2%.

Personal characteristics include age, urban-rural household registration and their own health. The average age of all samples was less than 35 years, of which the youngest was 20 years old and the oldest was 52 years old. In order to make the data more effective and eliminate the interference of data on the model, this paper takes the logarithm of the age samples. The samples are mainly young and middle-aged people, of which 756 are aged 26–35, accounting for 43.8%, and 582 are married women aged 36–45, accounting for 33.7%. The sample of married women who own urban household registration accounts for the majority. The urban household registration is set at 1, and the rural household registration is set at 0. In this paper, the self-rated health is taken as the health condition variable. Health as a variable is set to 0 when in good health and 1 when in bad health. Through descriptive statistics, we can see that the health condition of married women who are engaged in family elderly care is better than those who are not engaged in family care. This is because the children who are not in good health cannot provide good care for their parents (in-laws).

Family characteristics include bargaining power variable, whether to care for children under 6 years old variable, the number of members in family variable and whether to live with parents or in-laws variable. Bargaining power, as an important basis of family resource allocation, plays an important role in exploring the impact of family elderly care on married women’s employment. According to the Nash bargaining model, the best family allocation is determined by the wage rate when other conditions are unknown. In this paper, the wage rate as the standard, compared with the simple income variable, has more family significance, 1 means that men’s wages are less than women’s wages, 2 means that men’s wages are more than women’s wages, equal wages of men and women are taken as the reference group, with 0 as the reference group. Generally, married women in families with high male wages are more likely to bear the responsibility of family elderly care. It can also be seen from Table 1 that married women caring for children under the age of 6 are unable to take care of the elderly due to insufficient energy. At the same time, women with a large family cannot take care of their parents (in-laws) due to insufficient energy. In addition, whether to live with the elderly is also an influencing factor, but this influence is uncertain because if the elderly have the ability to take care of themselves, they do not need care and can help married women, on the contrary, they need their children to take care of them.

People with living parents (in-laws) are more likely to be engaged in family care for the elderly, while parents in need of care have no effect on their children’s employment decision-making. This variable only affects their children’s care responsibility to indirectly affect their employment. The number of brothers and sisters will affect married women’s care activities for the elderly. The family care responsibility with more brothers and sisters can be slightly reduced, because the caregivers’ brothers and sisters can share part of the care responsibility, but the number of brothers and sisters will not have an impact on employment. In this paper, the above two variables with correlation and exogenous characteristics are selected as instrumental variables to control endogeneity.

If there are multiple collinearity problems among the explanatory variables used, the estimated results may be biased because of the pseudo regression in the linear regression model. In this paper, we use variance inflation factor (VIF) to avoid the occurrence of pseudo regression. This method can determine whether there is a correlation between explanatory variables. The core idea of this method is to compare the ratio of variance in the case of multicollinearity between explanatory variables with that in the case of no multicollinearity. The larger the value of VIF, the more serious the collinearity between explanatory variables. When the VIF is lower than 10, the collinearity between explanatory variables does not exist. According to the test results in Table 2, the VIF between the explanatory variables in this paper is lower than 10, and the average of VIF is lower than 5, which indicates that the explanatory variables selected in this paper are correct, and there is no collinear problem between the variables.

## 3. Empirical Results and Analysis

### 3.1. Model Selection

In order to select the most suitable model, this paper assumes that there are several possibilities, and then carries out the screening test one by one. The results of model screening are shown in Table 3. Columns (1) and (2) are the mixed OLS results, columns (3) and (4) are the fixed effects (FE) results, and column (5) is the random effects (RE) results. The rationality of model (5) can be verified by analyzing the estimation results. Among them, columns (1) and (3) do not add the year dummy variable which can reflect the time characteristics, and columns (2)–(5) add the time dummy variable to control the time effect. In order to identify the influence of time on whether to participate in work, this paper uses the likelihood ratio test estimation results of columns (1)–(4). The test results show that whether this model uses the mixed OLS or fixed effect, there is a time effect, indicating that the linear model established in this paper has a time effect, which has a certain influence on whether to find a job. It can be seen from column (5) of Table 3 that when the Wald test was used to test the joint significance of years for random effects, the *p* value of rejecting the original hypothesis at the significance level of 5% is 0.0477, indicating the existence of a time effect. To sum up, the linear model established in this paper has a time effect. It can be seen that time affects the participation in work. In order to avoid a deviation, we should choose the model to control the time effect, that is, adding the time dummy variable into the model.

The individual effect is a kind of individual behavior difference that has to be ignored due to the limitation of the sample size when establishing the model. This kind of individual behavior difference will have an impact on the results, so the individual effect should be identified. In order to determine whether there is an individual effect, that is, whether the model chooses the mixed OLS in column (2) or fixed effect in column (4), the Wald test obtains F(1185, 526)= 1.63, Prob>F=0.0000, rejects the original hypothesis, indicating that there is an individual effect. At the same time, the Hausman test is used to judge whether to use the random effect, and the *p* value of accepting the original hypothesis is 0.1456, which shows that the random effect of adding the time dummy variable to analyze the influence of “whether to care for the elderly” on “whether to work” has a very good fitting estimation effect. Since the results of column (2) and column (5) are similar, in order to further illustrate the robustness of the random effects in the above analysis, we further use the B-P test and likelihood ratio to test the estimation results of the mixed OLS and random effects. The *p* value of rejecting the original hypothesis at the level of 1% is obtained by both the B-P test and the likelihood ratio test, which indicates that it is reasonable to use the random effect to estimate the influence of “whether to care for the elderly” on “whether to work”.

The result of column (5) shows that the estimated coefficient of the variable whether to care for the elderly is significantly negative at the 5% level, indicating that caring for the elderly will hinder work. At the same time, the binary variable with “whether to work” being 0,1. In order to test the robustness of the estimation results in column (5), the panel logit model is used for regression analysis. The regression results are shown in column (6). The results show that the estimation coefficient of “whether to care for the elderly” is still significantly negative at the level of 5%, which means that the probability of hindering the work of caring for the elderly is significantly higher than that of other situations, indicating that caring for the elderly has a significant negative impact on “going to work”, and this is consistent with the expected results.

### 3.2. Endogenous Problems

When using the random effect model to estimate the impact of “whether to care for the elderly” on “whether to work”, there will be endogenous problems due to the two-way causal relationship. To solve this problem, this paper selects the number of brothers and sisters and whether parents (in-laws) are alive as instrumental variables (IV) for regression, and the estimated results are shown in Table 4. The estimation coefficient of the important explanatory variable “whether to care for the elderly” in the estimation results of the first stage Generalized Space Two Stage Least Square (GS2SLS) Method in column (2) and the two-stage GS2SLS in column (3) is significantly negative at the 1% level, which is consistent with the estimation results of column (1) only using the random effect model (RE, which is significantly negative at the 5% level), indicating that caring for the elderly will hinder work. This result is consistent with the previous analysis, which indicates that the estimation results are robust. The results of other explanatory variables are also consistent with expectations, such as wage rate has a significant positive correlation with work, which shows that the perspective of bargaining power is reasonable and correct. The urban household registration has a greater probability of women working than the registered residence in the rural areas. The women with a large family size will have greater resistance to work and the women who live with their parents (in-laws) are more likely to take care of the elderly, so care activities are less likely to affect their work.

### 3.3. The Difference Analysis of Bargaining Power

As an important resource distribution activity in the family, the family elderly care activities can be analyzed by using the bargaining model. The stronger the bargaining power, the more family members can control the distribution mode. In order to investigate whether bargaining power affects married women’s employment, this paper divides the sample into three groups, and uses wage as an analysis index when other conditions are unknown, see Table 5. Using GS2SLS regression, the estimated coefficients of the samples with male bargaining power equal to that of female and the samples with male bargaining power higher than that of female are not significant, which indicates that caring for the elderly by this kind of family does not affect whether they work, while the estimated results of caring for the elderly with male bargaining power lower than that of female are significantly negative at the 5% level, which indicates that caring for the elderly by this kind of family has a significant negative impact on employment. This is because women’s bargaining power means that their total employment time is higher than that of family labor time, and they tend to have higher positions. The higher their positions are, the greater their responsibilities are. Care activities can only occupy a small part of their life time. The contradiction between caring for the elderly and their work is greater, and their working time is longer, thus squeezing the time of elderly care.

After ordinary regression, we use instrumental variables to control endogeneity to obtain more accurate results. The estimated results in Table 5 and Table 6 together show that in families where men’s bargaining power is lower than women’s bargaining power, taking care of the elderly can significantly hinder the employment of married women. The biggest difference between the two estimates is that taking care of the elderly by the female sample of the bargaining power of men is higher than that of women without considering endogeneity can promote employment, but the influence of caring for the elderly after considering endogeneity becomes an obstacle to employment. However, the influence of this sample group is not significant and has little influence on the results. This gap is also based on the fact that the number of samples may be different due to the lack of numerical value, but the results are more accurate and reasonable after controlling the endogeneity. It can be seen that the stronger bargaining power of women in the family, the greater the hindrance of caring for the elderly activities due to their participation in work. Women with strong bargaining power have stronger working ability, so it is difficult to balance the life between work and family when time is limited. Therefore, in this case, in order to achieve the result of cost optimization, the responsibility of family care for the elderly cannot be borne by women alone, so we can choose the formal care mentioned above.

## 4. Conclusions

According to the statistical yearbook in recent years, the aged-dependency ratio, which is in an upward trend year by year, reached 16.8% in 2018. At the same time, the ageing population also affects all aspects of the family. With the increase of the aged-dependency ratio year by year, the responsibility of children’s care is also increasing year by year. Based on the latest data of the CHNS, this paper uses OLS and the instrumental variable method to analyze the whole sample and bargaining power difference, and concludes that family elderly care poses an obstacle to married women’s employment, and the responsibility of elderly care in female families with strong bargaining power will significantly hinder their participation in employment. In the face of this situation, this paper puts forward public policy suggestions from two aspects: the introduction of policy and policy preference. In the face of this situation, this paper puts forward the following public policy suggestions:

(1) Take active pension policy. At present, the reduction of the number of children of the elderly weakens the ability of family care. At the same time, taking care of the elderly is not only the responsibility of children, but also part of the responsibility of the government for the basic living conditions of the elderly. The government should actively play a leading role in providing for the aged. First of all, we should establish the awareness of formal care of the whole society and change the misunderstanding of community care and institutional care. At the same time, we should increase financial investment, improve the pension service system, strengthen a series of security systems, such as social pension insurance and minimum living security, vigorously promote urban and rural community pension service, strengthen and stabilize the construction of pension nursing staff, reasonably allocate the post structure of pension nursing staff, continue to increase the training of pension service talents, implement relevant subsidies and other supporting policies, meet the spiritual and cultural needs of the elderly, so that all the elderly can have a sense of support, a sense of dependence, a sense of happiness and a sense of security. At present, some cities have launched a new mode of family care beds for the aged to promote the integrated development of community home care and institutional care. Under the background of the gradual deepening of population aging and the “comprehensive two child” policy, the burden of family members to care for the elderly will be further increased. Therefore, it is not enough to rely only on the role of the government. We should give full play to the role of market allocation. For example, the previous pilot work is completed under the joint efforts of the government and the market. On the basis of ensuring the welfare nature of public pension institutions, we should moderately introduce market competition mechanisms, mobilize and integrate available resources, promote the combination of public and private pension institutions, accelerate the formation of medium and high-end pension service centers, and meet a new pattern of elderly care of the multi-level and diversified care needs of all kinds of elderly people.

(2) Protecting women’s employment rights. The status of contemporary women is relatively rising. Women not only take the responsibility of family care, but also participate in work in pursuit of economic independence to realize their self-worth. However, it also brings the problem of balancing family and work. According to the analysis results, the stronger bargaining power of women, the greater the impact of family responsibilities on their work, and women’s participation in family care activities squeeze their working hours. On the one hand, it needs the husband and wife in the family to shoulder the responsibility of taking care of the elderly, and the traditional concept of “male dominating the outside and female dominating the inside” needs to be changed to create a good and harmonious family atmosphere. The state needs to introduce more laws and regulations to support women’s employment and safeguard their legitimate employment rights and interests. At the same time, we need to strengthen the gender perspective in the formulation of the old-age security system, include the awareness of gender equality in the old-age policy to safeguard the rights and interests of female caregivers, and incline to women in the formulation of supportive policies, so that female caregivers can better balance the relationship between work and family, and let women realize their economic independence and life autonomy.

(3) Give full play to the role of insurance. In addition to recognizing the disease risk and disability risk of the elderly, we should start with the income risk of the elderly and pay attention to their living conditions and personal needs from the source. This requires that the role of insurance security should be brought into play. At the same time of coordinating social endowment insurance, the awareness of social insurance should be improved, and the enterprise annuity system and commercial endowment insurance should be promoted to meet the multi-level and diversified living needs of the elderly. Commercial endowment insurance is the representative of family endowment insurance. It has a very good complementary effect with social endowment, and is a kind of pre-control awareness of risks. At present, the insurance companies have launched relevant insurance products, consumers should pay attention to screening when buying. At the same time, for the poor elderly who are an important group in poverty alleviation, the government must find a way out for these groups under the targeted poverty alleviation policy, and through the joint efforts of society and individuals, most elderly people can achieve poverty alleviation.

## Figures and Tables

**Table 1:** Descriptive statistics of the variables.

Variable	Mean Value	Standard Deviation	Minimum Value	Median	Maximum Value
Employment	0.826	0.379	0.000	1.000	1.000
Whether to care for the elderly	0.462	0.499	0.000	0.000	1.000
Wage rate	0.664	0.821	0.000	0.000	2.000
Age	3.544	0.225	2.773	3.555	4.007
Urban-rural household registration	0.695	0.461	0.000	1.000	1.000
Health	0.094	0.292	0.000	0.000	1.000
Whether to care for children under 6 years old	0.361	0.480	0.000	0.000	1.000
Number of members in family	4.350	1.760	1.000	4.000	16.000
Whether to live with parents (in-laws)	0.434	0.496	0.000	0.000	1.000

**Table 2:** Test results of the variance inflation factor.

Variable	VIF	1/VIF
Number of members in family	1.84	0.543970
Whether to live with parents (in-laws)	1.79	0.559037
Age	1.23	0.813052
Whether to care for children under 6 years old	1.12	0.892095
Urban-rural household registration	1.12	0.893279
Health	1.01	0.994118
Average of VIF	1.35	

**Table 3:** Estimation results of the model selection.

Variable	(1)	(2)	(3)	(4)	(5)	(6)
	OLS	OLS	FE	FE	RE	Panel Logit
Whether to care for the elderly	−0.054 ***	−0.043 **	−0.040	−0.047	−0.044 **	−0.496 **
	(−2.86)	(−2.26)	(−1.02)	(−1.21)	(−2.29)	(−2.28)
Wage rate	0.059 ***	0.066 ***	0.017	0.016	0.052 ***	0.600 ***
	(5.38)	(5.93)	(0.83)	(0.78)	(4.64)	(4.41)
Age	0.018	0.011	0.039	0.044	0.013	0.164
	(0.40)	(0.25)	(0.43)	(0.47)	(0.29)	(0.33)
Urban-rural household registration	0.080 ***	0.078 ***	−0.033	−0.033	0.069 ***	0.688 ***
	(3.90)	(3.77)	(−0.62)	(−0.63)	(3.24)	(3.05)
Health	0.042	0.034	0.058	0.056	0.034	0.408
	(1.36)	(1.09)	(1.08)	(1.03)	(1.15)	(1.18)
Whether to care for children under 6 years old	−0.001	−0.004	−0.008	−0.008	−0.006	−0.065
	(−0.03)	(−0.21)	(−0.24)	(−0.23)	(−0.30)	(−0.30)
Number of members in family	−0.026 ***	−0.022 ***	−0.000	0.003	−0.019 ***	−0.191 **
	(−3.73)	(−3.21)	(−0.01)	(0.14)	(−2.66)	(−2.53)
Whether to live with parents (in-laws)	0.072 ***	0.063 ***	−0.018	−0.023	0.057 **	0.546 **
	(2.97)	(2.60)	(−0.38)	(−0.45)	(2.34)	(2.05)
Constant	0.770 ***	0.735 ***	0.723 **	0.674 *	0.801 ***	2.228
	(4.65)	(4.04)	(2.14)	(1.85)	(4.76)	(1.23)
Individual effect	No control	No control	Control	Control	Control	Control
Time effect	No control	Control	No control	Control	Control	Control
*n*	1728	1728	1728	1728	1728	1728
R^2^	0.044	0.054	0.007	0.019	—	—

Note: ***, **, * represent 1%, 5%, 10% significance level, respectively. The values in brackets of columns (1)–(5) in the table are t values, the same as in the following table. The values in brackets of columns (6) in the table are z values (because logit regression uses maximum likelihood estimation).

**Table 4:** Estimation results of endogeneity.

Variable	(1)	(2)	(3)
	RE	RE + IV (One Stage)	RE + IV (Two Stage)
Whether to care for the elderly	−0.044 **	−0.081 ***	−0.081 ***
	(−2.29)	(−2.95)	(−2.95)
Wage rate	0.052 ***	0.051 ***	0.051 ***
	(4.64)	(3.96)	(3.96)
Age	0.013	0.039	0.039
	(0.29)	(0.72)	(0.72)
Urban-rural household registration	0.069 ***	0.044 *	0.044 *
	(3.24)	(1.74)	(1.74)
Health	0.034	0.032	0.032
	(1.15)	(0.91)	(0.91)
Whether to care for children under 6 years old	−0.006	−0.026	−0.026
	(−0.30)	(−1.11)	(−1.11)
Number of members in family	−0.019 ***	−0.017 **	−0.017 **
	(−2.66)	(−2.11)	(−2.11)
Whether to live with parents (in-laws)	0.057 **	0.050 *	0.050 *
	(2.34)	(1.74)	(1.74)
Constant	0.727 ***	0.691 ***	0.691 ***
	(3.99)	(3.13)	(3.13)
*n*	1728	1325	1325

Note: ***, **, * represent 1%, 5%, 10% significance level, respectively.

**Table 5:** Estimation results of bargaining power difference (without considering endogeneity).

Variable	The Bargaining Power of Men is Lower than that of Women	The Bargaining Power of Men is Higher than that of Women	The Bargaining Power of Men is Equal to that of Women
Whether to care for the elderly	−0.080 **	0.004	−0.023
	(−2.16)	(0.13)	(−0.81)
Number of samples	370	389	969

Note: ** represent 5% significance level, respectively.

**Table 6:** Estimation results of bargaining power difference (considering endogeneity).

Variable	Men’s Wages are Lower than Women’s	Men’s Wages are Higher than Women’s	Men’s Wages are Equal to Women’s
Whether to care for the elderly	−0.196 ***	−0.017	−0.063
	(−3.61)	(−0.36)	(−1.57)
Number of samples	284	312	729

Note: *** represent 1%, significance level, respectively.

## Data Availability

Not applicable.
